# Supplementary material for: Pneumococcal nasopharyngeal carriage in Indonesia infants and toddlers post-PCV13 vaccination in a 2+1 schedule: A prospective cohort study
Source: PLoS One. 2021 Jan 26;16(1):e0245789. doi: 10.1371/journal.pone.0245789 (PMC7837470; doi:10.1371/journal.pone.0245789)
Supplement: S1 Table — (DOCX) [file pone.0245789.s001.docx]

S1 Table. Vaccine type serotype distribution by age

| Age | | | Serotype | | | | | | | | Total |
| --- | --- | --- | --- | --- | --- | --- | --- | --- | --- | --- | --- |
|  |  |  | 6A/6B | 19F | 23F | 14 | 3 | 19A | 1 | 5 |  |
| 2 months | | | | | | | | | | | |
|  | Control group | n | 1 (1/9) | 1 (1/9) | 1 (1/9) | **4 (4/9)** | 2 (2/9) | 0 (0/9) | 0 (0/9) | 0 (0/9) | 9 |
|  | Vaccine group | n | **3 (3/7)** | **3 (3/7)** | 0 (0/7) | 1 (1/7) | 0 (0/7) | 0 (0/7) | 0 (0/7) | 0 (0/7) | 7 |
| 4 months | | | | | | | | | | | |
|  | Control group | n | 5 (5/23) | **6 (6/23)** | 5 (5/23) | 4 (4/23) | 1 (1/23) | 2 (2/23) | 0 (0/23) | 0 (0/23) | 23 |
|  | Vaccine group | n | **11 (11/23)** | 4 (4/23) | 6 (6/23) | 2 (2/23) | 0 (0/23) | 0 (0/23) | 0 (0/23) | 0 (0/23) | 23 |
| 12 months | | | | | | | | | | | |
|  | Control group | n | **16 (16/38)** | 7 (7/38) | 6 (6/38) | 5 (5/38) | 2 (2/38) | 2 (2/38) | 0 (0/38) | 0 (0/38) | 38 |
|  | Vaccine group | n | **11 (11/30)** | 3 (3/30) | **11 (11/30)** | 4 (4/30) | 1 (1/30) | 1 (1/30) | 0 (0/30) | 0 (0/30) | 30 |
| 18 months | | | | | | | | | | | |
|  | Control group | n | **20 (20/40)** | 4 (4/40) | 8 (8/40) | 5 (5/40) | 0 (0/40) | 1 (1/40) | 1 (1/40) | 1 (1/40) | 40 |
|  | Vaccine group | n | **6 (6/19)** | 5 (5/19) | 3 (3/19) | 2 (2/19) | 2 (2/19) | 0 (0/19) | 0 (0/19) | 1 (1/19) | 19 |
