# Supplementary material for: Pneumococcal nasopharyngeal carriage in Indonesia infants and toddlers post-PCV13 vaccination in a 2+1 schedule: A prospective cohort study
Source: PLoS One. 2021 Jan 26;16(1):e0245789. doi: 10.1371/journal.pone.0245789 (PMC7837470; doi:10.1371/journal.pone.0245789)
Supplement: S2 Table — (DOCX) [file pone.0245789.s002.docx]

S2 Table. Non-vaccine type serotype distribution by age

| Age | | | Serotype | | | | | | | | | | | | | | | | | | | | | | | | | | | N |
| --- | --- | --- | --- | --- | --- | --- | --- | --- | --- | --- | --- | --- | --- | --- | --- | --- | --- | --- | --- | --- | --- | --- | --- | --- | --- | --- | --- | --- | --- | --- |
|  |  |  | 15B/15C | 6C/6D | 15A/15F | 16F | 23A | 13 | 11A/11D | 35F/47F | 34 | 42/35A/35C | 23B | 33C/10C/10F | 10A | 35B | 24A/24F/24B | 22F/22A | 17F | 39 | 21 | 20 | 38/25F/25A | 18A/C/F | 37/33F/33A | 31 | 40/7C/7B | **untypeable** | |  |
|  |  |  |  |  |  |  |  |  |  |  |  |  |  |  |  |  |  |  |  |  |  |  |  |  |  |  |  | cpsA (+) | cpsA (-) |  |
| 2 months | | | | | | | | | | | | | | | | | | | | | | | | | | | | | | |
|  | C | n | 1 | **2** | 1 | 1 | 1 | 0 | 0 | **3** | 0 | 1 | 0 | 0 | 0 | 0 | 0 | 0 | 0 | 0 | 0 | 0 | 1 | 1 | 0 | 0 | 0 | 0 | 1 | 13 |
|  |  | % | 7 | **15** | 7 | 7 | 7 | 0 | 0 | **23** | 0 | 7 | 0 | 0 | 0 | 0 | 0 | 0 | 0 | 0 | 0 | 0 | 7 | 7 | 0 | 0 | 0 | 0 | 7 | 100 |
|  | V | n | **4** | 1 | 1 | **3** | 0 | **3** | 1 | 0 | 1 | 0 | 1 | 0 | 0 | 1 | 0 | 1 | 0 | 0 | 0 | 0 | 0 | 0 | 0 | 0 | 0 | 0 | **3** | 20 |
|  |  | % | **20** | 5 | 5 | **15** | 0 | **15** | 5 | 0 | 5 | 0 | 5 | 0 | 0 | 5 | 0 | 5 | 0 | 0 | 0 | 0 | 0 | 0 | 0 | 0 | 0 | 0 | **15** | 100 |
| 4 months | | | | | | | | | | | | | | | | | | | | | | | | | | | | | | |
|  | C | n | **5** | 2 | **3** | 1 | 1 | **3** | 2 | 0 | 2 | 1 | 0 | 0 | 1 | 0 | 1 | 0 | 1 | 1 | 1 | 0 | 0 | 1 | 0 | 0 | 0 | 2 | 1 | 29 |
|  |  | % | **17** | 7 | **10** | 3 | 3 | **10** | 7 | 0 | 7 | 3 | 0 | 0 | 3 | 0 | 3 | 0 | 3 | 3 | 3 | 0 | 0 | 3 | 0 | 0 | 0 | 7 | 3 | 100 |
|  | V | n | **10** | 1 | 2 | **3** | **3** | 1 | **3** | 1 | 1 | 0 | 1 | 0 | 1 | 0 | 0 | 1 | 0 | 0 | 0 | 1 | 0 | 0 | 0 | 0 | 0 | **4** | 1 | 34 |
|  |  | % | **29** | 6 | 6 | **9** | **9** | 3 | **9** | 3 | 3 | 0 | 3 | 0 | 3 | 0 | 0 | 3 | 0 | 0 | 0 | 3 | 0 | 0 | 0 | 0 | 0 | **12** | 3 | 100 |
| 12 months | | | | | | | | | | | | | | | | | | | | | | | | | | | | | | |
|  | C | n | 5 | 5 | 0 | 0 | **6** | **6** | 3 | 2 | 4 | 0 | 1 | 0 | 0 | 0 | 0 | 0 | 0 | 0 | 1 | 1 | 0 | 0 | 0 | 0 | 0 | 2 | 0 | 36 |
|  |  | % | 14 | 14 | 0 | 0 | **17** | **17** | 8 | 5 | 11 | 0 | 3 | 0 | 0 | 0 | 0 | 0 | 0 | 0 | 3 | 3 | 0 | 0 | 0 | 0 | 0 | 5 | 0 | 100 |
|  | V | n | **8** | **4** | 0 | 1 | 2 | 0 | 0 | 1 | 1 | 2 | 1 | 0 | 2 | 3 | 0 | 0 | 1 | 0 | 0 | 0 | 1 | 1 | 0 | 0 | 0 | **15** | 2 | 45 |
|  |  | % | **18** | **9** | 0 | 2 | 4 | 0 | 0 | 2 | 2 | 4 | 2 | 0 | 4 | 7 | 0 | 0 | 2 | 0 | 0 | 0 | 2 | 2 | 0 | 0 | 0 | **33** | 4 | 100 |
| 18 months | | | | | | | | | | | | | | | | | | | | | | | | | | | | | | |
|  | C | n | 4 | 1 | 2 | 1 | 2 | 2 | 2 | 0 | 2 | 0 | 0 | 0 | 0 | 0 | 1 | 0 | 0 | 1 | 1 | 1 | 0 | 0 | 1 | 0 | 0 | **6** | **10** | 37 |
|  |  | % | 11 | 3 | 5 | 3 | 5 | 5 | 5 | 0 | 5 | 0 | 0 | 0 | 0 | 0 | 3 | 0 | 0 | 3 | 3 | 3 | 0 | 0 | 3 | 0 | 0 | **16** | **27** | 100 |
|  | V | n | 9 | 0 | 1 | 0 | 2 | 2 | 2 | 1 | **11** | 1 | 0 | 0 | 3 | 0 | 2 | 0 | 1 | 0 | 0 | 1 | 0 | 0 | 0 | 1 | 1 | **12** | **12** | 62 |
|  |  | % | 14 | 0 | 2 | 0 | 3 | 3 | 3 | 2 | **18** | 2 | 0 | 0 | 5 | 0 | 3 | 0 | 2 | 0 | 0 | 2 | 0 | 0 | 0 | 2 | 2 | **19** | **19** | 100 |

**C = Control Group; V = Vaccine Group**
