# Supplementary material for: Pneumococcal nasopharyngeal carriage in Indonesia infants and toddlers post-PCV13 vaccination in a 2+1 schedule: A prospective cohort study
Source: PLoS One. 2021 Jan 26;16(1):e0245789. doi: 10.1371/journal.pone.0245789 (PMC7837470; doi:10.1371/journal.pone.0245789)
Supplement: Attachment — Submitted filename: renamed_bed1d.docx [file pone.0245789.s003.docx]

**COMMENTS – Manuscript PONE-D-20-14898**

Title: “Dynamic changes in nasopharyngeal pneumococcal serotype before and after pneumococcal comjugate vaccine administered on a 2+1 schedule”

**Important note: This review pertains only to ‘statistical aspects’ of the study and so** **‘****clinical aspects’ [like medical importance, relevance of the study,** **‘clinical significance and implication(s)’ of the whole study, etc.] are to be evaluated [should be assessed] separately/independently. Further please note that any ‘statistical review’ is generally done under the assumption that (such) study specific methodological [as well as execution] issues are perfectly taken care of by the investigator(s). This review is not an exception to that and so does not cover clinical aspects {however, seldom comments are made only if those issues are intimately / scientifically related & intermingle with ‘statistical aspects’ of the study}. Agreed that ‘statistical methods’ are used as just tools here, however, they are vital part of methodology [and so should be given due importance].**

**COMMENTS:** Study design is fairly simple; however, I have certain questions/doubts: In ‘Methods’ section, ‘Study design and participants’ subsection you said “Control group members were not immunized for PCV and lived in Central Lombok, while vaccine group members lived in West or East Lombok and received PCV13 immunization” which is O.K., but later you say “We use consecutive sampling method”, how is that done? What exactly you want to convey?

There are no comments on ‘Sample collection’ and ‘Laboratory procedures’ because they are beyond my area of knowledge. ‘Data analysis’ again is very simple [like simple study design]. You say ‘the Kolmogorov-Smirnov test was performed when some assumptions of chi-square were not satisfied’. The question is ‘which assumptions (of chi-square) were not satisfied?’ [note that Kolmogorov-Smirnov test is only a goodness-of-fit test; whereas chi-squared is a goodness-of-fit test as well as a test of association]

Since the ‘Article Type’ is ‘Clinical Trial’ it is expected to give details of required sample size estimation. Even ‘allocation’ procedure is desirable to be describe adequately. As said in ‘Methods’ section of abstract it is actually the ‘prospective cohort study’, but it is still desirable to discuss about ‘required sample size estimation’. I am not sure regarding important ‘clinical significance’ and ‘implication(s)’ of this study and so in my opinion it is vital to assess the study in this context by subject expert. ‘What the study contributes?’ is always the important question.

‘Study limitations’ mentioned are not very clear. As said earlier ‘Study design’ as well as ‘Data analyses’ are simple and therefore, not much to comment.
