# Supplementary material for: Pneumococcal nasopharyngeal carriage in Indonesia infants and toddlers post-PCV13 vaccination in a 2+1 schedule: A prospective cohort study
Source: PLoS One. 2021 Jan 26;16(1):e0245789. doi: 10.1371/journal.pone.0245789 (PMC7837470; doi:10.1371/journal.pone.0245789)
Supplement: Attachment — Submitted filename: Response to Reviewers.docx [file pone.0245789.s004.docx]

Dear Dr./Mr./Ms.

Thank you for giving us the opportunity to submit a revised draft of our manuscript titled **Pneumococcal nasopharyngeal carriage in Indonesia infants and toddlers post-PCV13 vaccination in a 2+1 schedule: a prospective cohort study** to *PLOS ONE*. We appreciate the time and effort that you and the reviewers have dedicated to providing your valuable feedback on our manuscript. We are grateful to the reviewers for their insightful comments on our paper. We have been able to incorporate changes to reflect most of the suggestions provided by the reviewers.

Here is a point-by-point response to the reviewers’ comments and concerns

| **Review** | | **Response to Reviewers** |
| --- | --- | --- |
| ***General Review*** | | |
|  | Please ensure that your manuscript meets PLOS ONE's style requirements, including those for file naming. The PLOS ONE style templates can be found at  https://journals.plos.org/plosone/s/file?id=wjVg/PLOSOne_formatting_sample_main_body.pdf and  <https://journals.plos.org/plosone/s/file?id=ba62/PLOSOne_formatting_sample_title_authors_affiliations.pdf> | Thank you for reminding us about this. We try to adjust our manuscript to PLOS ONE’s style requirements |
|  | While revising your submission, please upload your figure files to the Preflight Analysis and Conversion Engine (PACE) digital diagnostic tool, <https://pacev2.apexcovantage.com/>. PACE helps ensure that figures meet PLOS requirements. To use PACE, you must first register as a user. Registration is free. Then, login and navigate to the UPLOAD tab, where you will find detailed instructions on how to use the tool. If you encounter any issues or have any questions when using PACE, please email PLOS at [figures@plos.org](mailto:figures@plos.org). Please note that Supporting Information files do not need this step. | Thank you for giving the suggestion. We have upload the figure files to PACE diagnostic tool and meet PLOS ONE requirements |
| ***Comments from Reviewer* 1** | | |
|  | This paper aims to assess changes of pneumococcal carriage pre and post PCV13 administration, as a 2+1 schedule, in Indonesia. The key findings were that PCV13 as a 2+1 schedule reduced VT serotype carriage and reduced carriage of 6A/B as dominant serotypes. Overall, the scientific background and rationale for the investigation being reported could be strengthened and given further specificity. Strength of rationale would benefit is lacking. Overall, there is insufficient information regarding methods (e.g., sample size calculation, inclusion / exclusion criteria). It would be helpful to include further details in the results, such as a flow chart of participants, and a tabular comparison of participant characteristics stratified by control and vaccine cohorts. Largely the strengths and limitations of the paper and unaddressed, and should be reflected upon. In addition to the strong suggestion of using the STROBE checklist for observational studies, specific comments are below: The study design is a prospective cohort – it would be of benefit to include this in the Title. | Thank you for pointing this out. We agree with this point. Therefore, we have added several information in introduction (study rationale), methods (sample size, inclusion and exclusion criteria), and results (flow chart, comparison between cohort groups). In addition, we also use the STROBE checklist. |
|  | **Title: Page 1:** This title is descriptive and would draw the attention of those working in infectious diseases epidemiology, vaccinology, public health, and microbiology. Those working in the pneumococcal field would be drawn in particular. I would recommend specifying which PCV is under investigation. Further, it would be helpful to include the study design in the title. For example, something like “Pneumococcal nasopharyngeal carriage in Indonesian infants and toddlers post-PCV13 vaccination in a 2+1 schedule: a prospective cohort study.” | Agree. We have, accordingly, revised the title to emphasize this point. |
|  | **Abstract Page 2 Methods**: Would suggest inclusion of a few more details in the Methods section, including how participants were recruited; how pneumococci were detected and isolated; how was dominance of serotypes determined; and what statistical methods were used for inferential analyses? | Thank you for pointing this out. We agree with this comment. Therefore, we have added the information in Abstract. |
|  | **Abstract Page 2 Methods:** Here and throughout the paper, would recommend using the term prevalence if carriage is reported as % in this paper, rather than proportion. A proportion can only have values from zero to one. | We agree with this and have incorporated your suggestion throughout the manuscript. |
|  | **Abstract Page 2 Methods:** Would recommend including the age eligibility of participants and any other broad inclusion criteria – e.g., this study recruited infants 2 months of age, followed until 18 months of age. | Thank you for pointing this out. We agree with this comment. Therefore, we have added the information in Abstract. |
|  | **Abstract Page 2 Results**: Would suggest including a summary key demographics (e.g., x% of the participants were female), and any differences in participant characteristics by control and vaccine groups. | Thank you for pointing this out. We agree with this comment. Therefore, we have added the information in Abstract. |
|  | **Abstract Page 2 Results:** Would suggest including units and % after each reported age and prevalence, i.e., “the [prevalence] of VT serotypes in control groups aged 2 months, 4, months, 12 months, and 18 months was 40.9%, 44.2%, 53.8%, and 54.3%, respectively. | Thank you for pointing this out. We agree with this comment. Therefore, we have added the information in Abstract. |
|  | **Abstract Page 2 Results:** How was the p value determined, with regard to the reported significant decrease in VT serotypes compared with NVT serotypes? Further, would recommend reporting the p value itself, rather than p<0.05. In addition, it would be expected that the decline in VT serotypes would be greater than any decline in NVT serotypes, given the introduction of PCV13. | Thank you for pointing this out. The p value determined from inferential analyses between cohort groups, with regard to the reported significant decrease in VT serotypes compared with NVT serotype. We agree with this comment. Therefore, we have revised the p value in Abstract. |
|  | **Abstract Page 3 Conclusion**: I am unsure that the stated conclusion can be drawn from what appear to be crude analyses. | Thank you for the concern. We realize that this study only an introduction study for further analysis in Indonesia. |
|  | **Introduction Page 3:** Here and throughout, references are not cited where required – e.g., a reference for the statement that pneumonia is the leading cause of death in children under five years old worldwide – could recommend McAllister et al The Lancet, 2018 available here: https://www.thelancet.com/journals/langlo/article/PIIS2214-109X(18)30408-X/fulltext. | We agree with this and have incorporated your suggestion throughout the manuscript. |
|  | **Introduction Page 3:** Would recommend editing “The most common cause of pneumonia is Streptococcus pneumoniae” to “A leading cause of pneumonia is Streptococcus pneumoniae”. | Thank you for your suggestion. We have revised the sentence based on the combination of your review and third reviewer. |
|  | **Introduction Page 3:** Would suggest rewording “PCV administration can change …may be relative unchanged”. PCV reduces carriage, and thereby transmission of VT pneumococci, providing both direct and indirect protection from common disease causing serotypes. As a PCV program matures, serotype replacement may occur. | Agree. We have, accordingly, revised the sentence. |
|  | **Introduction Page 3 and 4:** Would suggest editing “In Bandung City, Indonesia, the pneumococcal carriage prevalence was 22.0% at the beginning of the study and increased to 68.4% after 10 months” to reflect that reported prevalence includes data from children recruited from both Bandung city and from a semi-rural area in Padalarang district ~25km from Bandung city – this will help provide some further context for the reader. | Agree. We have, accordingly, revised the sentence. |
|  | **Introduction Page 4:** In the paragraph describing the recommendations of the WHO with regard to schedules, it would be useful to include the information provided on page 13 as to where and why the WHO recommends different schedules. This will help provide background and information as to the current study setting. | Agree. We have, accordingly, modified the sentence. |
|  | **Introduction Page 4:** To provide some further specificity, would recommend indicating that “the goal of this study is to assess the effectiveness of PCV13” rather than just PCV; and including the age of healthy children; similarly, would recommend indicating the age of children, e.g., “… in healthy children aged 2 – 18 months in Indonesia.” | Agree. We have, accordingly, revised the sentence. |
|  | **Introduction:** were there any prespecified hypotheses? | Thank you for your concern. We have added the hypotheses in the last sentence of the introduction in manuscript |
|  | **Methods Page 4, Study Design and Participants:** The description of the study design, indication of location, and time frame of study were very clear. Further, the overt statement of ethical approval for the study is important, and described clearly. | Thank you for pointing this out. |
|  | **Methods Page 4/5, Study Design and Participants:** Some further details describing the setting and location would be welcome – for example, the population size, whether access to healthcare is universal, and rates of pneumonia among children aged under 5 | Thank you for pointing this out. We have added the information as your suggestion. |
|  | **Methods, Study Design and Participants Page 5:** Some key information regarding participants would be of value to include. For example, the eligibility criteria, sources and methods of participant recruitment, and methods of follow-up. Were control and vaccine groups matched? It appears that they may be age matched – where there any other matching criteria if relevant? | Thank you for pointing this out. We have added the information as your suggestion. |
|  | **Methods, Study Design and Participants Page 5**: The paper states that a consecutive sampling method was used, i.e., one in which every subject meeting inclusion criteria is selected until the required sample size is achieved. However, no sample size justification / calculation is provided, and neither are inclusion or exclusion criteria. Therefore it is unknown if the sample size provides sufficient power for this study. Further, if “healthy” children were recruited, what were the exclusion criteria – i.e., how was “healthy” determined? Were temperatures assessed and any child with a fever excluded? Were children with comorbidities, AOM, respiratory tract infections, taking antibiotics excluded? If a recruited child became unwell over the period they aged from 2 months to 18 months were they excluded? | Thank you for your suggestion. We think the better sampling method stated is convenience sampling. We have added additional information as required. |
|  | **Methods** – would recommend a section that describes and defines all variables. That is, define clearly, all outcomes, exposures, predictors, potential confounders, and effect modifiers as relevant. For example, “VT carriage was defined as carriage of serotypes 1, 3, 4, 5, 6A, 6B, 7F, 9V, 14, 19A, 19F, 18C, and 23F”. | Thank you for the recommendation. We have describes all variables. |
|  | **Methods, Sample collection, page 5:** would recommend specifying the swab material – presumably flocked nylon, as opposed to just flocked. | Thank you for the recommendation. We have specifying the swab material. |
|  | **Methods, Sample collection, page 5:** would recommend reviewing and referencing Satzke et al, Vaccine 2013 available here: https://www.clinicalkey.com.au/#!/content/playContent/1-s2.0-S0264410X13011742?returnurl=https:%2F%2Flinkinghub.elsevier.com%2Fretrieve%2Fpii%2FS0264410X13011742%3Fshowall%3Dtrue&referrer=https:%2F%2Fpubmed.ncbi.nlm.nih.gov%2F24331112%2F with regard to microbiological methods. It is important to cite this reference regarding the methods of NP sampling being in accordance with WHO guidelines. | Thank you for the recommendation. We have reviewing and referencing the source. |
|  | **Methods, Laboratory procedures, page 6:** How was the dominant serotype determined? | Thank you for pointing this out. We have added the information in the manuscript. |
|  | **Methods, Laboratory procedures, page 6:** What kind of blood was used in the blood agar plates? | Thank you for pointing this out. We have added the information in the manuscript. |
|  | **Methods, Data analysis page 6:** Was it possible to type all isolates? Were there any instances where an isolate was detected, but typing was not possible due to biological reasons or technical issues? If so, how was this missing data handled? | Thank you for pointing this out. We have added the information in the manuscript. |
|  | **Methods, Data analysis page 6:** It is unclear why participants with multiple carriage were assigned either VT or NVT only. Where participants carry both VT and NVT, it should be possible to record an observation of carriage for both VT and NVT serotypes. | Thank you for pointing this out. We agree this comment. Therefore, we have revised the information in the manuscript. |
|  | **Methods, Data analysis page 6:** It is unclear how the statistical methods employed address the stated goal of assessing vaccine effectiveness of PCV13 as a 2+1 schedule in health Indonesian children. It seems crude analysis only has been undertaken. Is this because the sample size was insufficient to permit more robust analysis, such as the standard 1 – odds ratio multiplied by 100, to determine VE? Further, would recommend that the analysis takes into account the significant differences in terms of participant characteristics by vaccine and control group, to determine the odds ratio for such analysis. | Thank you for this suggestion. It would have been interesting to explore this aspect. However, in the case of our study, it seems slightly out of scope because we only assessed the changing of pneumococcal colonization in nasopharynx, we can’t measure the antibody level or decreasing of disease burden. |
|  | **Methods, Data analysis page 6:** It is important to describe any efforts made to reduce any potential sources of bias – these may arise through selection processes, measurement processes, and differences in groups. Please could it be stated whether the microbiologists undertaking laboratory procedures were blinded to participant cohort and demographics? | Thank you for pointing this out. We have added additional information regarding this concern. |
|  | **Methods, Data analysis page 6:** Following the previous point, it is strongly recommended, that the two groups, i.e., vaccine and control, have participant demographics summarized via standard methods (i.e., categorical variables by counts and percentages, continuous variables by mean and standard deviation or median and interquartile range, as appropriate to the distribution) and then compared using appropriate methods. This is because it is important to determine if control and vaccine groups were different at baseline – differences in outcome may be due to differences at baseline, rather than the exposure of interest, and this should be considered. | Thank you for pointing this out. We have added additional information regarding this concern. |
|  | **Methods:** Which software was used for databases, and for descriptive and inferential analyses? | Thank you for pointing this out. We have added additional information regarding this concern. Data from case report forms and laboratory was entered to Microsoft Excel 365.  Datasets were imported and statistical analyzed using Graphpad Prism 8. |
|  | **Results: demographic characteristics, page 7:** Please include a flow chart of participants | Thank you for pointing this out. We have added a flow chart of participants (figure 1.) |
|  | **Results: demographic characteristics, page 7:** A summary of the participant characteristics by control and vaccine groups is essential. Would strongly recommend including this in tabular form, along with relevant comparisons. It would be of benefit to report characteristics such as sex distribution across groups, and any differences in characteristics by group, along with a statistical comparison. | Thank you for pointing this out. We have added additional information regarding this concern. |
|  | **Results: demographic characteristics, page 7:** The counts and percentages reported with regard to nutritional status and exposure to cigarette smoke by vaccine and control group appear to be quite different. Both of these factors have been shown in previous studies to be associated with pneumococcal carriage. For this reason, it is important to compare participant demographics by cohort group, and then adjust as necessary in inferential analyses. | Thank you for pointing this out. We have added additional information regarding this concern.  (The differentiation at baseline may leads to different outcomes. Inferential analyses using the chi-square test were performed to compare data between control and vaccine group.  Statistical significance was assigned at p &lt; 0.05. To reduce selection bias, intention to treat analysis was performed) |
|  | **Results: demographic characteristics, page 7:** Recommend replacing table 1 with a comparison of characteristics by cohort group, then undertaking formal analyses of association of factors associated with carriage, and using the results to determine effectives of PCV13. | Thank you for pointing this out. We have added table 1 with a comparison of characteristics by cohort group and undertaking formal analyses of association of factors associated with carriage, and using the results to determine effectives of PCV13. |
|  | **Results:** The proportion of S. pneumoniae, page 8: Here, and throughout, as in the Abstract, suggest replacing “proportion” with prevalence, and including a 95% confidence interval. | We agree with this and have incorporated your suggestion throughout the manuscript. |
|  | **Results: The pattern of S. pneumoniae serotype colonization, page 9:** Suggest moving “Multiple colonization is colonization by more than one ….during the entire study.” to Methods, in a section on Variables. | Agree. We have revised as your suggestion. |
|  | **Results: The pattern of S. pneumoniae serotype colonization, page 10:** This paper states that “After vaccination with PCV13, we expected that VT serotypes would be replaced by NVT serotypes or become negative, and did not expect that NVT serotypes would be replaced by VT or there would be no colonization by VT. “ Any such statements could usefully be reworded as pre-specified, hypotheses and included in the Introduction. However, the duration of this study is very short (16 months) which is may not be sufficient to for serotype replacement in a vaccine naïve population to emerge. | Agree. We have revised as your suggestion. |
|  | **Results: The pattern of S. pneumoniae serotype colonization, page 10:** This paper refers to “subjects with negative colonization chang[ing] to VT’ etc. It would be of value to describe carriage as observations. For example, “We observed participants in the control group who were non-pneumococcal carriers at age 2 months to have a higher prevalence of VT carriage at age 12 months, compared with participants in the vaccine group who were non-pneumococcal carriers at age 2 months.” | Thank you for pointing this out. We have added additional information regarding this concern. |
|  | **Discussion, page 10:** Recommend beginning the discussion with a summary of the key results along with reference to the study objectives. For example, “this study aimed to assess the effectiveness off of PCV13 …. We found ….” | Agree. We have revised as your suggestion. |
|  | **Discussion, page 11:** This paper states that “This means that PCV13 administration on a 2 + 1 schedule did not prevent an increase in pneumococcal colonization.” It is not clear whether this study has a sufficient sample size, or statistical methods that were undertaken, that support this statement. | Thank you for pointing this out. We have added additional information regarding this concern. |
|  | **Discussion, page 11:** This paper states that “These findings prove that there are other factors beyond PCV vaccination which affect pneumococcal colonization in the nasopharynx of infants and children…” This study does not examine factors associated with pneumococcal carriage. As such, it does not provide evidence to support this statement. However, many previous studies have investigated factors associated with pneumococcal carriage, including Fadlyana et al Pneumonia, 2018 (available here https://link.springer.com/article/10.1186/s41479-018-0058-1) which describes factors associated with pneumococcal nasopharyngeal carriage in young children living in Indonesia. Further, given it has been recognised that factors other than vaccination affect pneumococcal carriage, it would be worthwhile conducting analyse that a) determine factors relevant to carriage in the current study sample and b) take such factors into account in analyses. | Thank you for pointing this out. We have added additional information regarding this concern. |
|  | **Discussion, page 12:** The paper reports that “PCV13 is expected the result in no colonization of VT S.pneumoniae serotypes or replacement by NVT serotype. Although a microsimulation model from Finland predicted elimination of VT carriage among vaccinated individuals within 5 – 10 years of PCV introduction (under the assumption of a 90% vaccine coverage with 50% vaccine efficacy against acquisition) (see Nurhonen et al PLOS ONE 2013 https://journals.plos.org/plosone/article?id=10.1371/journal.pone.0056079), other evidence suggests that vaccine impact from transmission models based on data from low carriage prevalence settings, may translate to high carriage prevalence settings. Further, any elimination of VT carriage is likely to occur in settings with a mature PCV program, rather than 16 months of PCV, as supported by the paper’s inclusion of reference to the PRIME finding that there is persistent carriage of VT even after more than three years of post-PCV13 administration. As such, it is inconsistent to expect “PCV13 vaccination …to result in no colonization of VT S.pneumoniae serotypes…” | Thank you for pointing this out. We have added additional information regarding this concern. |
|  | **Discussion, page 13:** I am unconvinced that this study provides evidence to support the statement that “two primary doses are not enough to reduce VT serotypes in nasopharyngeal colonization.” | Thank you for your concern. We agree that this study cannot provides enough evidence to support the statement. Therefore, we try to find other sources to support the statement. |
|  | **Discussion, page 13:** The paper states that a “booster dose will only be effective if it is after two primary doses.” It should be noted that this is likely context dependent. For example, in a setting with a mature PCV program, it may be possible to move to a 1+ 1 schedule, as the UK has done. See Flasche et al PLOS Mid, 2015 (https://journals.plos.org/plosmedicine/article?id=10.1371/journal.pmed.1001839) and Choi et al PLOSMed, 2019 (https://journals.plos.org/plosmedicine/article?id=10.1371/journal.pmed.1002845). | Thank you for pointing this out. However, the statement were not from our study but from PRIME study. We only try to emphasize the results |
|  | **Study limitations, page 14:** I agree that a limitation of this study is the difficulty in determining duration of serotype acquisition – more frequent swabbing would be necessary to achieve this. Other limitations should also be acknowledged and reflected upon, including: the lack of generalizability beyond other healthy children; the differences between control and vaccine groups; reflections upon the sampling method; if the sample size is of sufficient size to provide power. | Thank you for your concern. We have added additional study limitations based on your suggestion. |
|  | **Conclusions, page 14:** The effectiveness of 2+1 studies is known. This study has not provided evidence for this, and does not assesses vaccines. | Thank you for the concern. We realize that this study only an introduction study for further analysis in Indonesia. |
|  | It may be of use to read and reflect upon the following articles: a. Wahl et al, Lancet Glob Health 2018 https://www.thelancet.com/journals/langlo/article/PIIS2214-109X(18)30247-X/fulltext b. Fadlyana et al Pneumonia, 2018 https://link.springer.com/article/10.1186/s41479-018-0058-1 c. Satzke et al, PLOS Med, 2015 https://journals.plos.org/plosmedicine/article?id=10.1371/journal.pmed.1001903 d. Greenberg et al, Clin Infect Dis 2016 https://academic.oup.com/cid/article/42/7/897/322584 e. Farida et al, PLOS ONE 2014 https://journals.plos.org/plosone/article?id=10.1371/jousrnal.pone.0087431 | Thank you for your recommendation. |
| ***Comments from Reviewer* 2** | | |
|  | This review pertains only to ‘statistical aspects’ of the study and so ‘clinical aspects’ [like medical importance, relevance of the study, ‘clinical significance and implication(s)’ of the whole study, etc.] are to be evaluated [should be assessed] separately/independently. Further please note that any ‘statistical review’ is generally done under the assumption that (such) study specific methodological [as well as execution] issues are perfectly taken care of by the investigator(s). This review is not an exception to that and so does not cover clinical aspects {however, seldom comments are made only if those issues are intimately / scientifically related & intermingle with ‘statistical aspects’ of the study}. Agreed that ‘statistical methods’ are used as just tools here, however, they are vital part of methodology [and so should be given due importance]. | |
|  | **COMMENTS**: Study design is fairly simple; however, I have certain questions/doubts: In ‘Methods’ section, ‘Study design and participants’ subsection you said “Control group members were not immunized for PCV and lived in Central Lombok, while vaccine group members lived in West or East Lombok and received PCV13 immunization” which is O.K., but later you say “We use consecutive sampling method”, how is that done? What exactly you want to convey? | Thank you for your concern. We think the better sampling method stated is convenience sampling. We have added additional information as required. |
|  | There are no comments on ‘Sample collection’ and ‘Laboratory procedures’ because they are beyond my area of knowledge. ‘Data analysis’ again is very simple [like simple study design]. You say ‘the Kolmogorov-Smirnov test was performed when some assumptions of chi-square were not satisfied’. The question is ‘which assumptions (of chi-square) were not satisfied?’ [note that Kolmogorov-Smirnov test is only a goodness-of-fit test; whereas chi-squared is a goodness-of-fit test as well as a test of association]  If only ‘0’ frequency in two cells [table-1] is the issue, I recommend to use RIDIT analysis [Bross IDJ. ‘How to use RIDIT analysis’. Biometrics, 1958; 14:18-38. There are many recent references, but they are on application results. This is (an original, old classic and) gives details on ‘how to use’ the technique]. | Thank you for pointing this out. There are two assumptions of chi-square which we concern, which are the expected value and the’0” frequency in the cells. The recommendation of alternative test is Kolmogorov-Smirnov test. However, if it doesn’t proper, we are looking forward for your further feedback. |
|  | Since the ‘Article Type’ is ‘Clinical Trial’ it is expected to give details of required sample size estimation. Even ‘allocation’ procedure is desirable to be describe adequately. As said in ‘Methods’ section of abstract it is actually the ‘prospective cohort study’, but it is still desirable to discuss about ‘required sample size estimation’. I am not sure regarding important ‘clinical significance’ and ‘implication(s)’ of this study and so in my opinion it is vital to assess the study in this context by subject expert. ‘What the study contributes?’ is always the important question. | This study is not the Clinical Trial because this study only observed the immunization given before by the ministry of Health. |
|  | ‘Study limitations’ mentioned are not very clear. As said earlier ‘Study design’ as well as ‘Data analyses’ are simple and therefore, not much to comment. |  |
| ***Comments from Reviewer* 3** | | |
|  | The authors present the results of a study investigating nasopharyngeal colonization rates with vaccine and non-vaccine type pneumococci following vaccination of infants with PCV in Indonesia, with the finding of lower VT colonization rates in the vaccinated group at 18 months. As mentioned by the authors, pneumonia and pneumococcal infections are a major cause of global mortality among infants and young children, and such studies are important to understand pneumococcal epidemiology and inform immunization programs in different regions. | |
|  | **Major Comments**  Revise/reorganize abstract and body of manuscript to clarify the relationship between the present study (which is referred to as a prospective cohort) and the clinical trial with vaccine and control groups. Was NP colonization a prespecified endpoint in a clinical trial, or was this a separate/ancillary cohort study of clinical trial participants? | Thank you for pointing this out. This study is a separate cohort study of clinical trial participants. |
|  | A table or figure depicting pneumococcal serotype distribution would be helpful, either in the main manuscript or as supplemental material | Agree. We will add a table in supplemental material |
|  | Limitations also appear to include relatively small sample size and single geographic area with distinct pneumococcal epidemiology, which may limit application to broader areas/populations | Agree. We have added your concern in our study limitations. |
|  | **Minor comments**  Title-spelling of pneumococcal conjugate vaccine | We agree with this. |
|  | Introduction-clarify if pneumococcus is the most common cause of bacterial pneumonia (viruses generally more common overall) | Thank you for your recommendation. We have revised the sentence. |

We look forward to hearing from you in due time regarding our submission and to respond to any further questions and comments you may have.

Sincerely,

Ari Prayitno
