# Supplementary material for: Pneumococcal nasopharyngeal carriage in Indonesia infants and toddlers post-PCV13 vaccination in a 2+1 schedule: A prospective cohort study
Source: PLoS One. 2021 Jan 26;16(1):e0245789. doi: 10.1371/journal.pone.0245789 (PMC7837470; doi:10.1371/journal.pone.0245789)
Supplement: Attachment — Submitted filename: Response to Reviewers [2] Ver 2.docx [file pone.0245789.s005.docx]

Dear Dr./Mr./Ms.

Thank you for giving us the opportunity to submit a revised draft of our manuscript titled **Pneumococcal nasopharyngeal carriage in Indonesia infants and toddlers post-PCV13 vaccination in a 2+1 schedule: a prospective cohort study** to *PLOS ONE*. We appreciate the time and effort that you and the reviewers have dedicated to provide us with insightful comments on our paper. We have edited the manuscript to address the suggestions provided by the reviewers.

Here is a point-by-point response to the reviewers’ comments and concerns

| **Review** | | **Response to Reviewers** |
| --- | --- | --- |
| ***Comments from Reviewer* 1** | | |
|  | This paper aims to assess changes of pneumococcal carriage pre and post PCV13 administration, as a 2+1 schedule, in Indonesia. The key findings were that PCV13 as a 2+1 schedule reduced VT serotype carriage and reduced carriage of 6A/B as dominant serotypes. The scientific background and rationale for the investigation being reported has been strengthened. The flow chart of participants is very useful, as is the description of the setting. The tabular comparison of participant characteristics stratified by control and vaccine cohorts is a welcome inclusion.  Further **specificity regarding methods** would be very helpful – for example **statistical methods and sample size calculation**. Of concern is **the appropriateness of the statistical methods to address the stated aim of the study.** Some specific comments and concerns are outlined below. | As suggested by the reviewer, we have added sample size calculation and further explanation of statistical methods. Some details based on your concern will be added below. |
|  | **Abstract Page 3 Conclusion:** I remain **unsure that the stated conclusion** can be drawn from these analyses. It may be more appropriate to edit to something akin to “This study describes lower prevalence of VT and 6A/6B serotypes among children who were PCV13 vaccinated compared with those who were unvaccinated” | We think this is an excellent suggestion and we really appreciate your concern. We have revised the conclusion based on your recommendation. |
|  | **Introduction:** With regard to the statement “The transmission of various pneumococcal infections can be prevented by the administration of the pneumococcal conjugate vaccine in infants under one year of age, I would recommend editing in line with the WHO definition of PCV vaccination, i.e., Two doses of PCV10 given before the age of 12 months, or one or more doses of PCV10 given at or after 12 months of age (see World Health Organization. Pneumococcal vaccines WHO position paper - 2012 - recommendations. Vaccine. 2012;30(32):4717-8. Epub 2012/05/25. doi: 10.1016/j.vaccine.2012.04.093. PubMed PMID: 22621828.] | As suggested by the reviewer, we have editing the line with WHO definition.  The revised text reads as follows on Introduction page 4:  “PCV10 dan PCV13 are licensed to reduce invasive pneumococcal disease (IPD). Two doses of PCV10 or PCV13 given before the age of 12 months at an interval of 4–8 weeks or more and one booster doses given between 9–15 months of age.” |
|  | **Introduction:** Could the authors please amend the section regarding previous research on NP carriage in children in Indonesia, to introduce the Bandung city study. This will help aid flow for the reader. | Thank you for pointing this out. We have revised the paragraph to help the reader. The revised paragraph will be found on Introduction page 4. |
|  | **Introduction:** The inclusion of pre-specified hypotheses in the introduction are very useful. I was unclear what the author’s meant by NVT serotypes would become “negative”. Please could this be revised? | Thank you for pointing this out. We have revised “negative” to “no colonization” on Introduction page 5 |
|  | **Methods, Study Design and Participants Page 5:** The authors have included some further details regarding sampling method, however no sample size justification / calculation has been provided. Please could the authors address this? | As suggested by the reviewer, we have added sample size calculation on Methods, Study Design and Participant page 6. |
|  | **Methods, Study Design and Participants:** Please could the authors provide some further clarity regarding eligibility, inclusion, and exclusion criteria? | The eligibility, inclusion, and exclusion criteria have been clarified on Methods, Study Design and Participants page 6 & 7. |
|  | **Methods, Data analysis page 6:** Could the authors please clarify how participants with multiple carriage were recorded? For example, did the authors record observations of positive to VT and NVT serotypes if participants carried both? Further, did the authors record observations of positive to serotype specific carriage where multiple serotypes were carried by the same participant? | Participants with multiple carriage will be recorded as the numbers of serotype. Therefore, the serotype changes will be observed for each serotypes. However, we will not show the detail serotype due to some considerations. The clarification has been inserted on Methods, Data Analysis page 9. |
|  | **Methods, Data analysis page 6:** It is unclear how the statistical methods employed address the stated goal of assessing vaccine effectiveness of PCV13 as a 2+1 schedule in health Indonesian children. It seems crude analysis only has been undertaken. Is this because the sample size was insufficient to permit more robust analysis, such as the standard 1 – odds ratio multiplied by 100, to determine VE? Further, would recommend that the analysis takes into account the significant differences in terms of participant characteristics by vaccine and control group, to determine the odds ratio for such analysis. It is not necessary to measure the antibody level or decreasing of disease burden to conduct such analysis. Some further clarity regarding how demographic characteristics were summarized would be of value. For example, “categorical variables were summarized by counts and percent, and continuous variables were summarized by median and (IQR).” | Due to small sample size and limited time, the vaccine effectiveness (VE) were not assessed in this study. However, we assessed the changes of nasopharyngeal colonization as the prior condition leading to IPD with or without PCV13 vaccination.  The clarification regarding how demographic characteristics were summarized have been inserted in the text as your suggestion on Methods, Data Analysis page 9. Furthermore, we have determine the odds ratio for participant characteristics. |
|  | **Methods.** The authors describe a prospective cohort study. Factors affecting recruitment and enrolment of participants into a prospective cohort study are unlikely to introduce selection bias. This is because in order for selection bias to occur, the selection has to be related to both exposure and outcomes. But in a prospective cohort study, participants are enrolled before the outcome of interest has occurred (in this case, carriage). Although enrolment may be related to exposure status, the prospective nature of the study makes it difficult to consider the outcome (carriage) as influencing enrolment. | We agree with the reviewer’s assessment. Accordingly, we have omitted the selection bias concept in this study. |
|  | **Results:** Useful to see a comparison of characteristics by cohort group. It would be useful to include analysis of association of factors associated with carriage, and using the results to determine effectives of PCV13 – this could be achieved by building logistic regression models to investigate the association of PCV13 vaccination with the outcome of interest (carriage) adjusting for potential confounders, and then estimating vaccine effectiveness using 1 – odds ratio x 100 | Thank you for your suggestion. We have try to build logistic regression models to investigate the potential confounders. However, the results show a very big number which tells us that the sample size need to be higher. Therefore, we did not include the logistic regression models in the manuscript. |
|  | **Discussion:** This paper states that “This means that PCV13 administration on a 2 + 1 schedule did not prevent an increase in pneumococcal colonization.” It is not clear whether this study has a sufficient sample size, or statistical methods that were undertaken, that support this statement. | We agree with the reviewer’s assessment. Therefore, we have omitted the statement and hold on the first statement of the paragraph which stated that, “The prevalence of *S. pneumoniae* increased with age in the control and vaccine groups, and there was no significant difference in either group in changes of *S. pneumoniae* colonization by age.” |
|  | **Discussion:** I remain unconvinced that this study provides evidence to support the statement that “two primary doses are not enough to reduce VT serotypes in nasopharyngeal colonization”. | We agree with the reviewer’s assessment. Accordingly, we have amended the statement on Discussion, page 17 |
|  | **Discussion, page 13:** The paper states that a “the booster dose will only be effective if it is after two primary doses and will not be effective after three primary doses.” The authors indicate that this statement is not from their current study, but from the PRIME study. In the paper cited (Whitney et al Pediatr Infect Dis J 2014), the authors state “overall booster doses are clearly beneficial for programs that use only 2 primary doses, but the clinical benefit of a booster dose remains uncertain for programs that achieve high coverage with 3 primary doses”. This is different from suggesting that a booster dose will only be effective after two primary doses and will not be effective after three primary doses. Effectiveness of booster doses and primary doses are context dependent. In a setting with a mature PCV program, it may be possible to move to a 1+ 1 schedule, as the UK has done. See Flasche et al PLOS Mid, 2015 (https://journals.plos.org/plosmedicine/article?id=10.1371/journal.pmed.1001839) and Choi et al PLOSMed, 2019 (https://journals.plos.org/plosmedicine/article?id=10.1371/journal.pmed.1002845). I would recommend some amendment to reflect the context specific nature of the effectiveness of the number of primary and booster doses. | As suggested by the reviewer, we have amended this section. We have omitted the statement of “the booster dose will only be effective if it is after two primary doses and will not be effective after three primary doses.”  The revised text reads as follows on Discussion, page 17:  “However, Whitney et al. [11] mentioned that the effectiveness between schedules only relevant in the early PCV program and context dependent. Furthermore, Flasche et al. [33] and Choi et al. [34] stated that it may even be sufficient to move to a 1+1 schedule in mature PCV program.” |
| ***Comments from Reviewer 2*** | | |
|  | Since most of the comments (at least major ones) made on earlier draft by me (and hopefully by other respected reviewers also as there were many) are attended positively/adequately, and, in my opinion, the manuscript is improved a lot. I feel that now the manuscript has achieved acceptable level of our journal. | Thank you so much! |
| ***Comments from Reviewer 3*** | | |
|  | With some additional clarification of the study design and characteristics of the study cohorts, several additional questions arise that should be addressed:  1. The authors now refer to the sampling method as a “convenience sample” and provide some information regarding the initial clinical trial. However key details regarding the selection methods are still lacking. Specifically:   a) How many participants were included in the original clinical trial? Recommend adding the numbers enrolled in the initial vaccine trial to the top of the flow diagram shown in Figure 1. b) Based on the first version of the manuscript and subsequent revision, it can be inferred that consecutive participants in the clinical trial were enrolled into the two cohorts based on geographic location/vaccine group assignment, however this is still not explicitly stated. Is this accurate? Please make selection methods clear and specific in the manuscript. c) Please include more details regarding the power analysis used to determine the size of the cohorts in this study. What endpoint(s) was the study powered for? This seems important in determining whether the study was sufficiently powered to support the authors' conclusions re: overall carriage rates. | Thank you for your concern.   a. The vaccine initially administered to 251 infants by Indonesian Ministry of Health in East and West Lombok. From them, we recruited 118 infants to our study. b. The participants enrolled into the two cohort groups based on the schedule of vaccination program conducted by Indonesian Ministry of Health (the vaccination schedule divided by the geographic location). The vaccination program started at West and East Lombok (we assigned as vaccinated groups), whereas the vaccination in Central Lombok conducted latter (we assigned as control groups). c. We use power of 80% in this study. The endpoint was to assess the changes of nasopharyngeal colonization with or without PCV13 administration. |
|  | 2. While additional demographic information is now included, the analysis does not take potential confounders into account, and is, as mentioned by others, a crude analysis. For example, the characteristics of the cohorts indicate a difference in rates of malnutrition between groups-while the authors state that baseline differences could affect outcome measures (and that there is a previously reported association between pneumococcal carriage and nutritional status), the statistical models do not account for such variables as covariates. Have the authors attempted to perform a multivariate analysis to investigate this? | Thank you for pointing this out. We have attempted to perform a multivariate analysis to investigate further. However, the results show a very big number which tells us that the sample size need to be higher. Therefore, we did not include the logistic regression models in the manuscript. |
|  | Minor comments:  **Page 4:** Where mentioned, provide a brief definition of serotype replacement and the general timeframe in which it typically occurs, for readers who may not be familiar with this phenomenon. | As suggested by the reviewer, we provide further explanation about serotype replacement definition. |

We look forward to hearing from you in due time regarding our submission and to respond to any further questions and comments you may have.

Sincerely,

Ari Prayitno
